# Essential role of endogenous prolactin and CDK7 in estrogen-induced upregulation of the prolactin receptor in breast cancer cells

**DOI:** 10.18632/oncotarget.16040

**Published:** 2017-03-09

**Authors:** Raghuveer Kavarthapu, Maria L. Dufau

**Affiliations:** ^1^ Section on Molecular Endocrinology, Eunice Kennedy Schriver National Institute of Child Health and Human Development, National Institutes of Health, Bethesda, MD 20892-4510, USA

**Keywords:** estrogen receptor, prolactin, prolactin receptor, estradiol, STAT5

## Abstract

Our early studies have shown that Estradiol (E_2_)/Estrogen Receptor α (ER) in a non-DNA dependent manner through complex formation with C/EBPβ/SP1 induced transcriptional activation of the generic hPIII promoter and expression of the Prolactin Receptor (PRLR) receptor in MCF-7 cells. Subsequent studies demonstrated effects of unliganded ERα with requisite participation of endogenous PRL on the activation of PRLR transcription. Also, EGF/ERBB1 in the absence of PRL and E_2_ effectively induced upregulation of the PRLR. In this study we have delineated the transcriptional mechanism of upregulation of PRLR receptor induced by E_2_ incorporating knowledge of the various transcriptional upregulation modalities from our previous studies. Here, we demonstrate an essential requirement of STAT5a induced by PRL via PRLR receptor which associates at the promoter and its interaction with phoshoERα S118. Knock-down of PRL by siRNA significantly reduced E_2_-induced PRLR promoter activity, mRNA and protein expression, recruitment of ERα to the complex at promoter, C/EBPβ association to its DNA site and productive complex formation at hPIII promoter. The specific CDK7 inhibitor (THZ1) that attenuates E_2_-induced ERα phosphorylation at S118 abrogated E_2_-induced PRLR promoter activation. Further studies demonstrated that E_2_ induced cell migration was inhibited by PRL siRNA and THZ1 indicating its dependence on PRL/PRLR and CDK7, respectively. Our studies have demonstrated the essential role of endogenous PRL and CDK7 in the upregulation of PRLR by E_2_ and provide insights for therapeutic approaches that will mitigate the transcription/expression of PRLR and its participation in breast cancer progression fueled by E_2_ and PRL via their cognate receptors.

## INTRODUCTION

Prolactin Receptor (PRLR) belongs to the class I cytokine receptor superfamily. The long form of PRLR has an extracellular domain, a single transmembrane domain and intracellular cytoplasmic domain which is required for signal transduction. Prolactin (PRL) hormone binds to PRLR with high affinity and mediates its actions predominantly through JAK-2/STAT5 signaling pathway and other pathways that involve MAPK and the participation of JAK2/c-SRC family kinases/focal adhesion kinase via phosphatidylinositol 3-kinase [1–3]. PRLR is well known to play an important role in the physiology of the human breast and in the etiology, progression breast cancer [4–6]. Also, substantial clinical and etiological evidence indicates that estrogen exposure promotes the development, progression and invasion of breast cancer. Its effects are mediated via estrogen receptors through a host of estrogen responsive genes [7–9], and through signaling pathways generate through non-genomic pathways [10]. ER-independent effects resulting from direct genotoxic insult of its metabolites have been also recognized [11]. An important connection has been established between ERα and PRLR where both liganded and un-liganded ERα were found to participate in PRLR regulation [12, 13].

Our previous studies demonstrated upregulation of transcription/expression of the PRLR gene by estradiol through its preferential utilized promoter PIII promoter (hPIII/hE1_3_), which is one of six promoters of the hPRLR gene with their corresponding alternative non-coding exons 1. Among these, in addition of the generic promoter 1/exon1 (hPIII/hE1_3_) which is also present in rat and mouse, the other five are human specific (promoters1-5/exon1-5, hE_N1-5_) [14–16]. Estradiol (E_2_) through activation of the hPIII promoter lacking an estrogen responsive element induce increases of PRLR non-coding exon-1 hE1_3_ mRNA transcripts, PRLR mRNA and protein. The hPIII promoter contains functional Sp1 and C/EBP sites that bind Sp1/Sp3 and C/EBPβ, respectively. Non-DNA bound ERα activated by E_2_ associates in a complex with Sp1 and C/EBPβ bound to their cognate elements. These in turn recruit coactivators and through epigenetic changes favor the recruitment of TFIIB, and Pol II for transcriptional induced expression of PRLR gene by E_2_ in MCF-7 breast cancer cells [12, 17].

In related studies we demonstrated that ERα preexist as homodimers even in absence of E_2_, and that a conformation change in ERα induced by E_2_ has an important role in its increased interaction with C/EBPβ and Sp1 [18].

**Figure 1:**
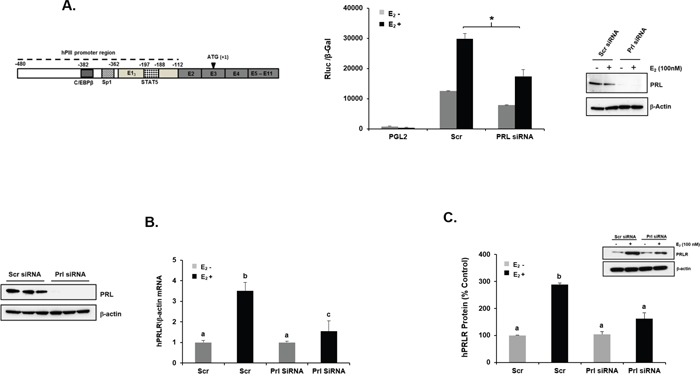
E2 induced upregulation of PRLR gene transcription/expression inhibited by knock-down of endogenous PRL **(A)** *Left*- Schematic representation of PRLR gene with the generic promoter hPIII (indicated in dotted line -480/-112) which includes the non-coding exon-1 (hE1_3_) required for promoter activity [15, 16] and also the DNA sites of relevant transcription factors within hPIII are indicated. In addition the common non-coding exon 2 and coding exons 3-11 are shown. *Right*- Effect of E_2_ (100 nM) on PRLR promoter activity in cells transfected with PGL2 construct (control) or wild type hPIII/hE1_3_ and with/without Scrambled (Scr) or PRL siRNAs. Results presented are relative luciferase activities (Rluc) normalized to the activities of co-transfected β-galactosidase. Asterisks (*) indicate statistically significant reduction in PRL siRNA treated group induced by E_2_ compared to Scr siRNA treated group induced by E_2_ (Student *t*-test; *P* < 0.01). Relative mRNA **(B)** and protein expression **(C)** levels of PRLR normalized by endogenous β-actin upon E_2_ induction in MCF-7 cells transfected with either Scr or PRL siRNAs. *Left*- Western blot of PRL protein expression in Scr siRNA and PRL siRNA transfected samples. Different superscript letters indicate significant differences (Tukey's multiple comparison test, *P* < 0.05). **(C)** *Right*- Western blot PRLR protein expression in Scr siRNA and PRL siRNA transfected samples (right). Results in this figure and below are reported as the mean ± SE of three independent experiments.

Our recent studies revealed upregulation of the PRLR induced by endogenous and exogenos prolactin (PRL) through its receptor in the absence of estradiol in MCF-7 and T47D-cells where unliganded-ERα, JAK2/STAT5, mitogen-activated protein kinase (MAPK) and PI3K pathways were found to have essential roles. Phospho- ERα associates with the C/EBPβ-SP1 as complex at the PRLR hPIII promoter. JAK2- activated STAT5a and b (phospho-forms) associate as tetramer of homodimers or heterodimers with a functional GAS element in the non-coding exon 1 (hE1_3_) corresponding to the hPIII promoter [13]. This exon was found in our previous studies to be required for the transcriptional activity of the hPIII promoter (Figure 1A, left) [15, 16]. Other recent studies have indicated a role for paracrine EGF via EGFR/ERBB1 independent of estrogen and prolactin in the transcriptional activation of PRLR gene expression. Unliganded ERα and STAT5b, which are both phosphorylated by EGF-induced activation of EGFR/ERRB1 through separate independent mechanisms, are both required in the PRLR activation of gene expression by EGF [19]. Of major relevance were our findings whereby interaction of ERα with STAT5 at the promoter was found to be required for association of phospho-ERα to the complex with consequent induction of the PRLR gene by PRL or EGF. These findings were indicative of STAT5 as stabilizing factor on unliganded ERα association to the complex at the PIII promoter.

Since PRLR and EGFR are expressed in breast tumors and STAT5 was found essential for unliganded ERα dimer association with the complex at the PRLR promoter [13, 19], we envisioned commonalities in the mechanism that participate in the E_2_/ERα induction of PRLR gene transcription/expression to that observed for PRL and EGF in MCF-7 cells. Thus, it was of much interest to apply our current knowledge to further studies on the intrinsic mechanism of upregulation of PRLR by E_2_/ERα. Phosphorylation of ERα is effectively induced by E_2_ at S118 in the AF1 domain via cyclin dependent kinase CDK7/cyclin H complex [20] and JAK2 derived signal transduction pathways, MAPK and PI3K, induced by endogenous PRL via PRLR could also contribute to ERα phosphorylation. Moreover, since other aspects of the PRL/PRLR/JAK2/STAT5 are of relevance in transcriptional activation of PRLR we proceeded to investigate their role in the E_2_/ERα upregulation of the PRLR. In this study we demonstrated the requirement of the participation of PRL/PRLR in transcriptional upregulation/expression of the PRLR induced by E_2_/ERα in breast cancer cells.

## RESULTS

### PRL is required for E_2_ induced PRLR transcription/ expression

In previous studies we determined that endogenous PRL which is present in breast cancer cells contributes to basal levels of PRLR expression in MCF-7 and T47D cells and these were magnified by addition of exogenous PRL. Also, in early studies we demonstrated that E_2_/ERα induces upregulation of PRLR transcription. We initiated studies to determine whether endogenous PRL action in breast cancer cells is required for E_2_ induced increase in PRLR gene expression. Cells cultured in the absence of E_2_ transfected with PRL siRNA showed marked depletion of endogenous PRL levels compared to control cells transfected with scrambled siRNA. Control cells (Scrambled siRNA) upon E_2_ stimulation showed significant increases in PRLR promoter (hPIII generic) activity (Figure 1A), PRLR mRNA (Figure 1B) and protein levels (Figure 1C) by E_2_ over basal. In contrast, minimal or non-significant increases of these parameter induced by E_2_ were observed in cells where endogenous PRL was depleted by PRL siRNA, (Figure 1). These findings indicate that endogenous PRL is required for the increases in PRLR induced by E_2_ in breast cancer cells.

### Role of STAT5 in the stimulation of PRLR by E_2_/ERα

**Figure 2:**
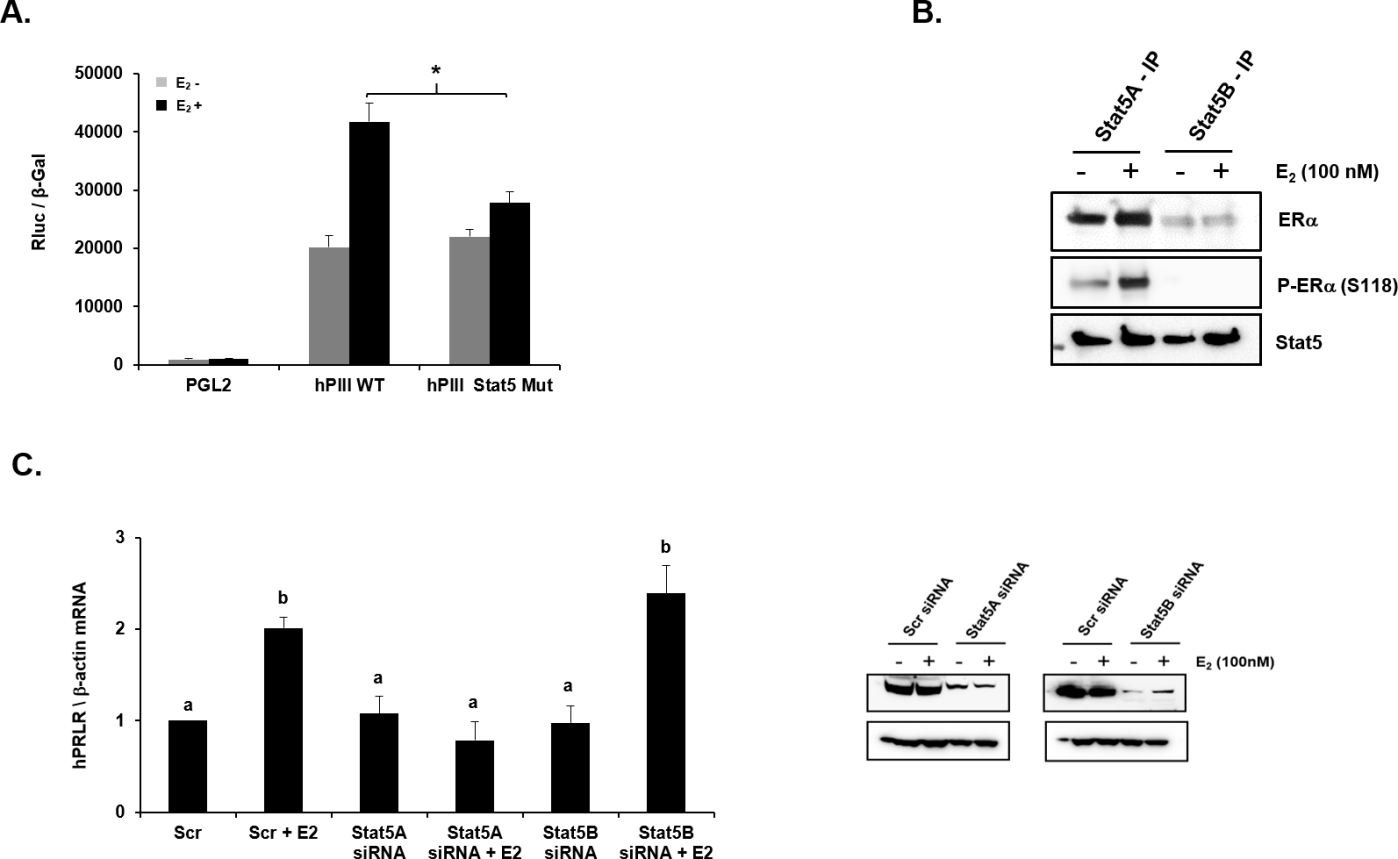
Role of STAT5 in E2-induced promoter activity and mRNA expression of PRLR **(A)** Effect of E_2_ on PRLR promoter activity of cells transfected with pGL2 vector (basal) or hPIII construct (WT) or hPIII construct with mutation in STAT5 binding site. Asterisk (*) indicate the statistically significant reduction in E_2_-induced cells transfected with hPIII construct with mutation in STAT5 binding site when compared with E_2_ induced cells transfected hPIII construct (WT) (Student *t*-test; *P* < 0.01). **(B)** Immuno-precipitation analysis showing interaction between ERα/pERα and STAT5a in MCF-7 cells treated in presence or absence of E_2_. **(C)** *Left-* Expression of PRLR mRNA in cells transfected with coding region of STAT5a siRNA or STAT5b siRNA or Scr siRNA. *Right-* Western blot showing knock-down of STAT5a and STAT5b by siRNA. Different superscript letters indicate significant differences (Tukey's multiple comparison test, *P* < 0.05).

Subsequent studies were directed to determine the mechanism of the PRL participation for the increase in PRLR induced by E_2_. In cells transfected with hPIII WT PRLR promoter E_2_ significantly stimulated the promoter activity while in cells transfected with hPIII with mutated STAT5 element, residing in non-coding exon-1 of the PRLR promoter, the activation by E_2_ was minimally present (Figure 2A). This indicated that PRL/PRLR through activation JAK2 and Stat5 is required for the E_2_/ERα stimulus of PRLR. Activated STAT5 induced by PRL independent of E_2_ associates with STAT5 DNA element to stimulate PRLR transcription [13]. In the case of the PRLR increase through E_2_ stimulation the findings indicate the requisite participation of PRL in STAT5 activation. Since in previous studies PRL-induced STAT5 activation in absence of E_2_ and ERα was found to interact with STAT5 in Re-ChIP assay [13], we initially investigated whether STAT5a or STAT5b associated with ERα. Immunoprecipitates of cells cultured in presence or absence of E_2_ with STAT5a and STAT5b antibodies revealed that only STAT5a interacts with ERα. Furthermore an association of phospho ERα at S118 with STAT5a was demonstrated (Figure 2B). In cultures where STAT5a or STAT5b was significantly depleted by their corresponding siRNAs (Figure 2C, right panel), E_2_ stimulation of PRLR mRNA expression was completely abolished in STAT5a depleted cultures while no effects were observed in the case of cells with depleted STAT5b (Figure 2C).

**Figure 3:**
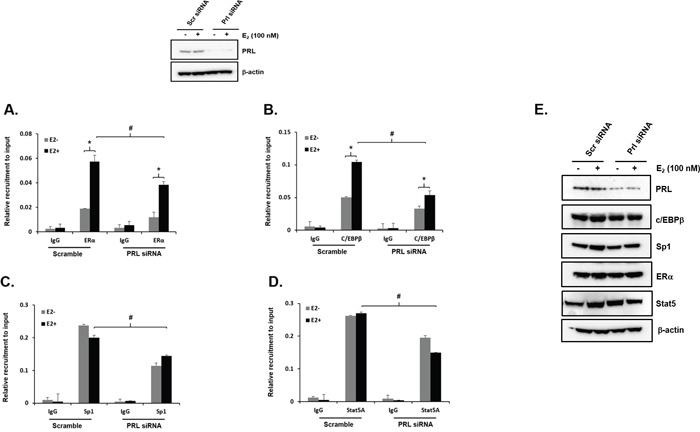
Effect of endogenous PRL knockdown on Recruitment of ERα, Sp1, C/EBPβ and STAT5a on to the PRLR promoter *Above-* Western blot of MCF-7 Cells transfected with Scr siRNA or PRL siRNA for 48 h subsequently incubated with E_2_ or ethanol (control) after serum starvation for 2 days showing depletion of endogenous PRL by siRNA vs Scr siRNA for A-D shown below. ChIP assay was performed using these cells to observe the recruitment of ERα **(A)**, C/EBPβ **(B)**, Sp1 **(C)** and STAT5a **(D)** onto the PRLR promoter. Asterisks (*) indicate statistically significant changes between E_2_ untreated and treated groups (Student-t test; *P* < 0.001). Hash (#) indicate statistically significant changes between Scr and PRL siRNA transfected groups E (Student-t test; *P* < 0.05). Western blot showing depletion of endogenous PRL by siRNA versus Scr siRNA, and unchanged levels of ERα, C/EBPβ, Sp1 and STAT5a expression in PRL depleted versus control cells.

We previously demonstrated that ERα associates in a DNA independent manner to C/EBPβ/Sp1 which in turn associate at their DNA sites at the hPIII promoter [18] and this was markedly enhanced by E_2_ treatment of cells with complex formation of E_2_/ERα.-C/EBPβ/Sp1 [12, 18] Studies on the association of these transcription factors to the hPIII promoter revealed that in cells with knock-down of endogenous PRL by specific siRNA (Left, Figure 3 Above), recruitment of ERα-induced by E_2_ was significantly decreased compared to controls with scrambled siRNA (Figure 3A) Also, the increased recruitment of C/EBPβ to the promoter induced by E_2_ in control cells was completely abolished by PRL knock-down (Figure 3B). Sp1 is constitutive associated to the hPIII promoter and E_2_ as in previous studies did not induce recruitment. However, it is apparent that in cells with PRL depleted Sp1 recruitment was markedly reduced and no significant differences were observed in presence or absence of E_2_ (Figure 3C). Recruitment of STAT5a, presumably induced by endogenous PRL/PRLR, in control cells was not influenced by E_2_ treatment. Consequently in cells with PRL knock-down STAT5a was significantly reduced and no differences were observed in presence or absence of estradiol (Figure 3D). The protein expression of these transcription factors were not influenced by the depletion of endogenous PRL (Figure 3E).

### Role of CDK7 in the E_2_/ERα induced PRLR transcription

**Figure 4:**
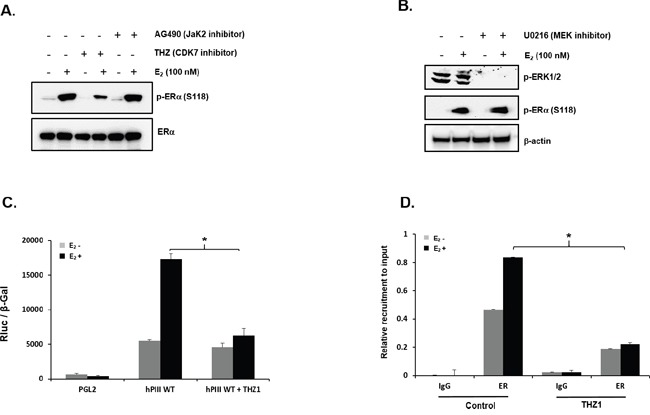
Role of CDK-7 in E2 induced promoter activity **(A)** Representative Western blot showing the phosphorylation status of ERα at serine 118 (S118) in MCF-7 cells in controls and after treatment with the CDK7 inhibitor, THZ1 (100 nM), or AG-490 (100 μM) for 3 h prior to E_2_ treatment for 30 min. Endogenous ERα and β-actin are used as loading controls. **(B)** Western blot showing effect of U0126 (10 μM) on phosphorylation status of ERα at S118 and ERK1/2 in cells cultured in presence or absence of E_2_ for 30 min following pre-incubation with or without inhibitor. β-actin is used as loading control. **(C)** Effect of CDK-7 inhibitor on E_2_ induced upregulation of hPIII transcriptional activity. MCF-7 cells transfected with PGL2 vector (control) or with hPIII promoter construct were pre-incubated with or without THZ1 (100 nM) inhibitor for 3 h prior to addition of E_2_ and further incubated for 18 h. Results presented are relative luciferase activities (RLuc) normalized to the activities of co-transfected β-galactosidase. Asterisks (*) indicate statistically significant changes between E_2_ untreated and treated groups (Student *t*-test; *P* < 0.001). **(D)** Effect of THZ1 (100 nM) treatment of cells as in **(C)** on E_2_-induced recruitment of ERα to the complex at the promoter assessed by ChIP assay.

Subsequent studies in MCF7 cells demonstrated phosphorylation of ERα at Ser 118 in a ligand-dependent manner by CDK7 [20] inhibited by the specific covalent CDK7 inhibitor THZ1 [21], while the AG490 (JAK2 inhibitor) or MEK inhibitor had no effect (Figure 4A and 4B). Further studies demonstrated that the CDK7 inhibitor completely abrogated the stimulation of hPIII PRLR promoter activity by E2 (Figure 4C). Moreover, THZ1 treatment of MCF7 cells caused complete inhibition of E_2_-induced recruitment of ERα to the hPIII promoter versus basal control (Figure 4D). These findings indicate that inhibition of E_2_-induced ERα (S118) phosphorylation by the CDK7 inhibitor contributed to the near complete inhibition of E_2_-induced PRLR promoter activation.

### Role of endogenous PRL and CDK7 in E_2_ induced cell migration

**Figure 5:**
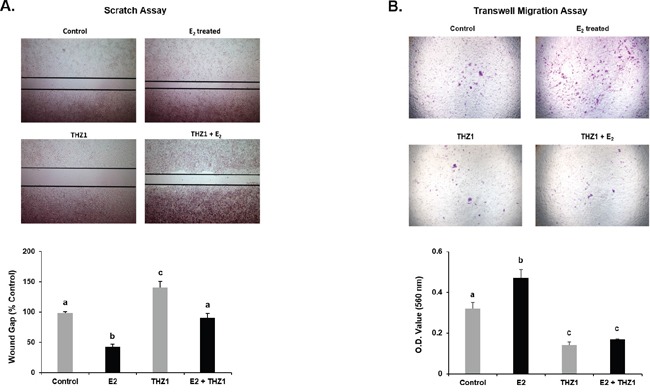
Effect of CDK-7 treatment on E2 induced MCF-7 cell migration by Scratch (A) and Trans-well (B) assays **(A)** MCF-7 cells pre-incubated with THZ1 (100 nM) for 3 h were subjected to scratch/wound healing assay to assess the E_2_ induced cell migration. After 48 h photographs were taken and wound width was calculated using ImageJ software. Representative images showing the wound width in control and treated groups. Graph showing the wound gap (percentage of control) in different groups. Different superscript letters indicate statistical significance between experimental groups (Tukey's multiple comparison test, *P* < 0.05). **(B)** Effect of THZ1 (100 nM) on migration induced by E_2_ assessed by Transwell assay following pre-treatment of MCF7 cells with THZ1 for three hours (see methods). *Below* Relative absorbance of lyzed cells detected on migrated membranes. Different superscript letters indicate statistical significance between experimental groups (Tukey's multiple comparison test, *P* < 0.05). Data represents the Mean ± SE from three independent experiments.

**Figure 6:**
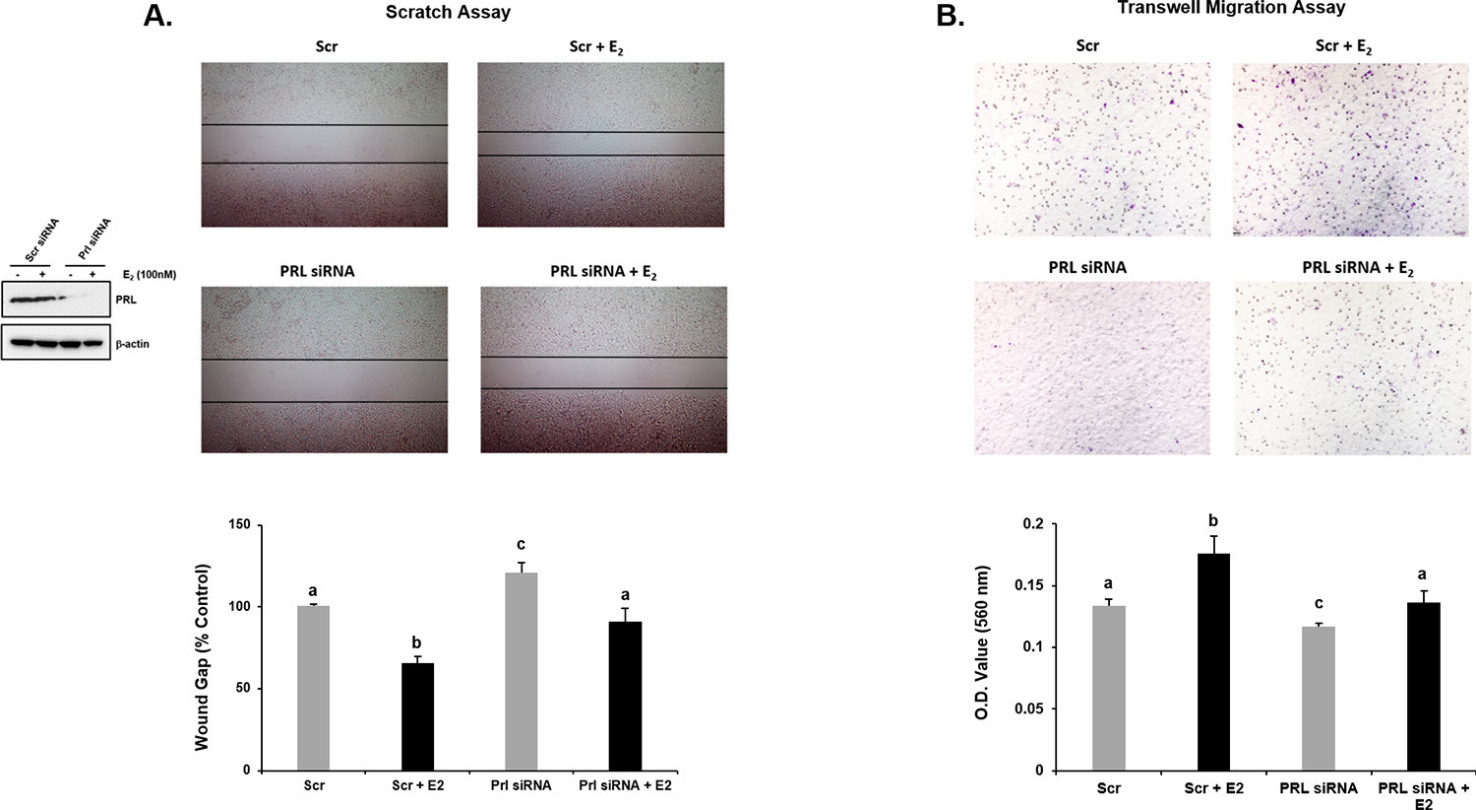
Effect of PRL knockdown on E2 induced MCF-7 cell migration by Scratch (A) and Trans-well (B) assays **(A)** *Left* siRNA and control treated with Scr siRNA were assessed for E_2_- induced cell migration. MCF-7 cells transfected with Scr or PRL siRNA were subjected to scratch/wound healing assay to assess the E_2_ induced cell migration. After 48 h photographs were taken and wound width was calculated using ImageJ software. Representative images showing the wound width in control and treated groups. Graph showing the wound gap (percentage of control). Different superscript letters indicate statistical significance between experimental groups (Tukey's multiple comparison test, *P* < 0.05). **(B)** Effect of PRL siRNA knock-down on migration induced by E_2_ assessed by Transwell assay. *Below* Relative absorbance of corresponding to lyzed cells present on migrate membranes. Different superscript letters indicate statistical significance between experimental groups (Tukey's multiple comparison test, *P* < 0.05).

To assess whether endogenous PRL and CDK7 have a role in E_2_-induced cell migration we performed wound healing/scratch assays in MCF-7 cells exposed to THZ1 inhibitor (Figure 5A) or PRL siRNA (Figure 6A), before E_2_ treatment in serum starved conditions. In untransfected (control) or scrambled (Scr) siRNA transfected cells, percentage wound gap was significantly narrower under E_2_ treatment than untreated controls. THZ1 treatments (Figure 5A) and knockdown of endogenous PRL by PRL siRNA (Figure 6A) resulted in increased wound gap in the cells with or without E_2_ induction. Similarly, the inhibitory role of the CDK7 inhibitor on E_2_-induced cell migration was demonstrated by Transwell assays (Figure 5B). Also, significant reduction of E_2_ induced cell migration was observed when endogenous PRL was knock-down by siRNA (Figure 6B). These results indicate that both endogenous PRL and CDK7 are involved in E_2_-induced cell migration in MCF-7 cells. The findings derived from migration studies are of much relevance since these resemble the essential role of PRL and CDK7 in PRLR upregulation induced by E_2_/ERα (Figures 1-5), and PRLR is require to mediate the actions of PRL.

## DISCUSSION

In this study we have analyzed the intrinsic mechanism of the PRLR upregulation induced by Estradiol via its ERα receptor previously recognized in our laboratory [12, 18]. We were prompted by our subsequent findings on the modalities of upregulation of the PRLR receptor by un-liganded ERα [13], and its independent regulation by Epidermal Growth through its receptor EGF/ERBB1 [19]. The finding of a functional GAS site in non-coding exon-1 [13] which is contained in the hPIII of the PRLR participating in both aspects of regulation provided fertile ground to expand our knowledge on the mechanism of E_2_/ERα.

In this study we demonstrate the essential role of endogenous PRL in the upregulation PRLR induced by E_2_/ERα with a requisite participation of STAT5a induced by PRL via PRLR. Phosphorylated STAT5a which associates with its functional element at hPIII interacts with non-DNA bound E_2_/ERα which in turn associates in a complex to Sp1 and C/EBPβ bound to their cognate DNA sites at the PRLR hPIII promoter. E_2_ favors ERα phosphorylation at S118 by CDK7 kinase, (Figure 4A) and greatly increase the recruitment of E_2_/ERα to the PRLR promoter over basal unliganded ERα and its association with pSTAT5a (Figure 2B). Phosphorylation of ERα at S118 is necessary for its association to the complex and its interaction with STAT5a. Inhibition by the specific CDK7 inhibitor, THZ1 markedly reduced the E_2_-induced ERα phosphorylation at S118, while the JAK2 inhibitor AG490 or MEK inhibitor U0219 which inhibits downstream JAK2 induced pathways [13] known to phosphorylate unliganded ERα at S118 and S167 had no effect (Figure 4A and 4B). THZ1 effectively abolished E_2_ induced ERα recruitment to the PRLR hPIII promoter (Figure 4D) and consequently caused a marked reduction of E_2_-induced PRLR transcription to control levels (Figure 4C). This clearly indicated the role of pERα (S118) and the essential requirement of CDK7 in the transcriptional upregulation of the PRLR receptor induced by E_2_ (Figure 4C).

**Figure 7:**
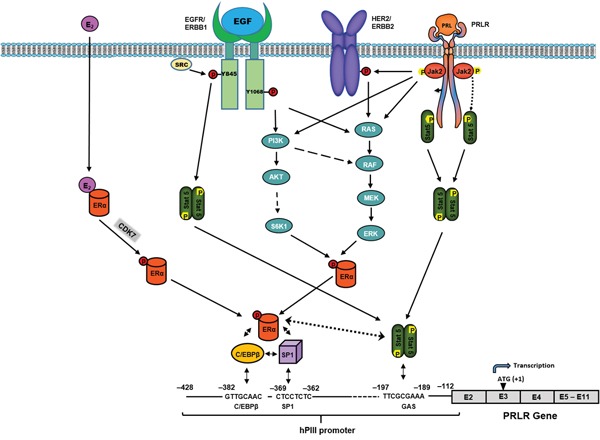
Mechanisms of the upregulation of hPRLR induced by E2/ERα revealed in this study and others defined in our previous studies [13, 19] Requirement of association of pERα (S118) induced by E2 via phosphorylation by CDK7 (non-DNA bound) and complex formation with C/EBP/Sp1 bound to their respective DNA elements at the hPIII promoter PRL/PRLR is essential for transcriptional activation of PRLR through activation of STAT5 which associates its site at the hPIII promoter and stabilizes the complex by association with ERα, a requirement for recruitment of coactivators, TFIIB and Pol II [12]. Inhibition of pERα (S118) by the CDK7 inhibitor causes abolition complex formation and of transcription/expression. The use of this inhibitor could provide an alternative efficacious adjuvant therapy to prevent inductive and progression effects of E_2_/ERα in breast cancer. Also, presented schematically are other previously described modalities on the up-regulation of PRLR via PRL/PRLR/JAK2/STAT5 per se, or through HER2 [13] transactivation by JAK2. In addition, are shown EGF functional effects via its cognate receptor ERBB1, independent of PRL/PRLR and E2 [19], on the activation of intrinsic receptor tyrosine kinase and signal transduction pathways participating in STAT5 and ERα phosphorylation/activation, respectively, -essential for PRLR gene transcription through hPIII promoter.

Endogenous prolactin is essential for activation of pSTAT5a which its presence at the DNA site is required for the recruitment and stability of the complex. This has been established in ChIP studies where PRL was knock-down by siRNA and recruitment of STAT5a was effectively reduced at its site in the promoter. In this instance a major reduction was observed on ERα and C/EBPβ induced by E_2_. In the absence of E_2_ significant reductions on ERα recruitment to hPIII (basal) (Figure 3A) was also observed in this study as in our previous studies on the unliganded ERα [13] with PRL knock-down, which are comparable to the basal values in this study (Figure 3A). This led us to propose a role for endogenous PRL/PRLR/STAT5 and of STAT5a interaction in the stability of the complex. This proposal has been demonstrated in this study where E_2_ recruitment of ERα to the complex was significantly reduced with PRL knock-down. In addition, basal and E_2_ stimulated C/EBPβ were similarly reduced (Figure 3A-3C). The efficiency of C/EBPβ recruitment to its DNA site was greatly enhanced when ERα/Sp1 complexes were preformed and DNA-bound Sp1 was the preferred interacting partner of ERα [18]. However, the reduction of Sp1 recruitment was not expected since it appeared to be associated to the complex constitutively and not shown to be induced by E_2_ in previous [12, 18] and this study Figure 3C. The absence of stabilization of the complex by reduction of STAT5a/ERα interaction could reduce the recruitment C/EBPβ and Sp1. In this regard studies where C/EBPβ was depleted with siRNA in cells treated in presence or absence of E_2_ a marked reduction of Sp1 association to its DNA site was observed when compared to control. Thus, the association of ERα to Sp1 seems to be important for recruitment of C/EBPβ to its site and in turn C/EBPβ and -Sp1 associations secure the stable recruitment of Sp1 and the overall complex formation (Figure 7).

The mechanism of upregulation of PRLR by E_2_/ERα defined in this study displays commonalties and specific differences with those described for unliganded ERα and EGF/EGFR [13, 19]. Although mechanistically all require STAT5 association with hPIII the specific STAT5 class participation which associates to hPIII reveal differences, STAT5a and b or specifically STAT5a, in both cases where involvement of prolactin was required and STAT5b in the case of EGF/EGFR participation. This may be related in part to specific requirements for their phosphorylation which is a requisite for their recruitment to its site at hPIII and/or presumably for their interaction with ERα. Also, differences in modality of phosphorylation of ERα have been indicated above and summarized in Table 1.

**Table 1:** Comparative summary of mechanisms of upregulation of PRLR induced by E_2_, PRL and EGF through liganded and un-liganded pERα and STAT5

Phosphorylation	E_2_	PRL*	EGF**
**ERα**	Cdk 7 (**S118)**	1) PRLR/JAK2/PI3K/MEK/ERK (**S118; S167**) 2) PRLR/JAK2/HER2-HER3/RAS/RAF/MEK1/2/ERK (**S118; S167**) 3) JAK2/HER2-HER3/ PI3K/AKT/S6K1 (**S118; S167**)	1) ERBB1 1068,1086 RAS/RAF/MEK/ERK (**S118**)2) ERBB1 1068, 1086PI3K/AKT/mTOR/ S6K1 (**S167**)
**STAT5**	PRL/PRLR/JAK2pSTAT5a	PRL/PRLR/JAK2pSTAT5a & pSTAT5b	EGF/ERBB1Y845 c-SRCpSTAT5b

* independent of E_2_; ** independent of E_2_ and PRL

ERα Serine (S) (phospho-position)

Recent studies using kinase inhibitors and gene editing demonstrated that triple-negative breast cancer cells are transcriptional dependent on CDK7. A cluster of genes in these cells were found to be exquisitely sensitive to the CDK7 inhibitor THZ1 [22]. In this regard, it is of interest that the inhibition of CDK7 which participates in the phosphorylation of ERα at S118 require for E_2_ induced upregulation of PRLR, also effectively inhibited the well-known induction of cell migration induced by E_2_ (Figures 5 and 6). Prolactin as E_2_ through similar mechanism has been reported to promote cell migration [23–25]. This was further established in this study by knock-down of endogenous PRL which resulted in inhibition of E_2_-induced cell migration. This indicated a dependence on PRL/PRLR on E_2_-induced cell migration and their mutual link to CDK7 participation (Figure 6). Targeting CDK7 kinase, which is known to regulate both transcription and the cell cycle and ERα phosphorylation with the THZ1 inhibitor was found to effectively inhibit the transcription of the PRLR and its contribution to cell migration induced E_2_/ERα in breast cancer cells. THZ1 treatment could provide an additional avenue singly or in combination with other inhibitory approaches targeting receptor function (PRLR [26], ERα [13]), HER2 [27], ERBB1 [19]) and/or signaling pathways (MAPK, PI3K, c-SRC [13, 19], ERBB1Tyrosine kinase, [19]) to effectively ablate transcription of PRLR and its contribution to breast cancer.

## MATERIALS AND METHODS

### Reagents and antibodies

Phenol-red free RPMI 1640 media and RPMI 1640-GlutaMAX were purchased from ThermoFisher Scientific. Charcoal/Dextran treated Fetal Bovine Serum (FBS) was obtained from Atlanta Biologicals. STAT5a, STAT5b, STAT5, ERα, β-actin antibodies and AG-490 (JAK2 inhibitor) were obtained from Santa Cruz Biotechnology. Phospho (p)-ERK1/2, and U1026 (MEK1/2 inhibitor) were from Cell Signaling and the CDK7 inhibitor, THZ1 hydrochloride from Med Chem Express. pERα (Ser118) antibody was obtained from EMD Millipore. PRLR antibody were obtained from Sigma-Aldrich. Human PRL antibody were obtained from National Hormone and Peptide Program, Harbor-UCLA Med. Ctr., Torrance, CA 90502.

### Cell culture and reporter gene assay

The MCF-7A2 (MCF-7) ER positive breast cancer cells (gift from E. Berleth, C. Roswell Park Cancer Institute, New York, NY) were maintained in RPMI 1640-GlutaMAX supplemented with 10% charcoal stripped FBS at 37°C in CO_2_ incubator. Cells were cultured in 24 well plates using phenol red-free RPMI media with 5% and 1% charcoal-treated FBS for 2 days at each serum concentration. The PRLR generic promoter (hPIII promoter/non-coding exon 1 [hE1_3_]) with reporter pGL2 gene construct (bp −480/−112) containing C/EBPβ, Sp1 sites in hPIII and putative STAT5 binding site in hE1_3_, and construct with the STAT5 site mutated, and pGL2 empty vector were used for transient transfection in MCF-7 cells using Lipofectamine 3000 reagent (ThermoFisher Scientific) as described previously [18]. Cells were treated with E_2_ (100 nM) in RPMI phenol red-free media for 18 h. To study the effect of CDK-7 inhibitor (THZ1) on the PRLR generic promoter activity, cells pre-incubated with THZ1 for 3 h prior treatment with 100 nM E_2_ and incubation for 24 h. Cells were harvested to the determine PRLR promoter activity using Brigh-Glo luciferase assay system (Promega). The E_2_ 100 nM dose used in the present studies was derived from our previous work demonstrating that addition of E_2_ (1-100 nM) to the cultures caused dose dependent increases in PRLR promoter activity, mRNA and protein [12].

### Cell-migration assays

### Wound healing/scratch assay

MCF-7 cells (5 x 10^4^ cells/well) were cultured in 6 well plates with phenol red free RPMI media containing 5% charcoal stripped FBS at 37°C in CO_2_ incubator. Then next day cells were transfected with 20 nM scrambled and PRL silencer select siRNAs using siPORTNeoFX reagent (Life Technologies) and grown in 1% charcoal stripped FBS till cells reach 90% confluent. Then cell monolayers were wounded by scratching with sterile 100 μL micropipette tips. Fresh medium containing 100 nM E_2_ was added every 12 h. After 48 h cells were photographed under phase contrast inverted microscope and cell migration was assessed by measuring migration distance (wound gap) using ImageJ Software from National Institutes of Health. To study the effect of THZ1 inhibitor on cell migration, cells were pre-incubated with the inhibitor for 3 h prior treatment with E_2_.

### Transwell assay

MCF-7 cells were cultured and transfected as indicated above and incubated in presence or absence of THZ1 inhibitor for 3 h. Transwell assay was performed using 6.5 mm inserts with 8 μm pore size polycarbonate membrane inserts (Cell Biolabs, Inc). Subsequently, 0.5 X 10^6^ cells in 300 μl of RPMI phenol red free were seeded on the top chamber and 500 μl RPMI containing 10% charcoal-treated FBS with or without E_2_ (100 nM) was applied to the lower chamber. Migration was assessed after incubation of culture plates at 37°C in a 5% CO2 incubator for 24-48 h. After removal of non-migrated cells from the upper chamber, membranes were fixed in methanol for 5 min. The cells that are migrated to the lower side of the membrane were stained with cell stain solution (crystal violet) provided in the kit for 5 min. The migrated cells were by visualized under a light microscopy and photograph at 5X magnification. Also, migrated cells were lyzed using 200 μl of extraction solution and the absorbance at 560 nm was recorded for indirect estimation of the number of migrated cells [28].

### Western blot analysis

Whole cell lysates from MCF-7 cells cultured in serum starved conditions in presence and absence of E_2_ (100 nM) or treated with U0126 (10 μM) or THZ1 (100 nM) inhibitors were extracted using RIPA lysis buffer (Thermo Scientific, Rockford, IL) in presence of 1x protease and Phosphatase inhibitor cocktail (Thermo Scientific). The protein samples were run on NuPAGE 4 -12% Bis-Tris gradient gels and transferred to nitrocellulose membranes using iBlot 2 (ThermoFisher Scientific). Membranes were blocked with 5% skimmed milk powder in phosphate buffer saline and incubated with different primary antibodies or β-actin (loading control) and washed and incubated with secondary antibody conjugated with HRP (Pierce). Immunodetection was performed using super-signal chemiluminescence system (Pierce).

### siRNA analysis

Silencer Select pre-designed validated siRNAs from Ambion (ThermoFisher Scientific) were used to knock-down the endogenous expression of PRL, STAT5a, STAT5b in MCF-7 cells. Scrambled siRNA was used as negative control. The MCF-7 cells were transfected with 20 nM siRNA using siPORTNeoFX or Lipofectamine RNAiMAX reagent (ThermoFisher Scientific) as described previously [18]. After 48 h of siRNA transfection cells were grown in 5% charcoal treated FBS for 1 day and 1% for additional 2 days. The cells were harvested for Chip assays and Western blots after treatment with E_2_ (100 nM) in serum free medium for 18 h. All the Silencer Select siRNA sequences used for RNAi interference are shown in Table 2.

**Table 2:** List of validated siRNAs used for gene knockdown and their sequence information

Gene	Sequence (Sense)
PRL	GCGAAUUCGAUAAACGGUATT
STAT5a	AUGGAUAUGUGAAACCACATT
STAT5b	CACCCGCAAUGAUUACAGUTT
Scrambled siRNA	AATTCTCCGAACGTGTCACGT

### Chromatin immunoprecipitation (ChIP) assay

MAGnify™ Chromatin Immunoprecipitation system from Invitrogen was used to perform ChIP assays according to the manufacturer's protocol as described previously [18]. The relative binding of Sp1, C/EBPβ and STAT5 proteins to their respective putative DNA binding sites on PRLR promoter was quantitatively evaluated by qRT-PCR of the precipitated DNA and input DNA using SYBER Green FAST Master Mix in an ABI 7500 Fast Real-Time PCR system. The primers used for amplification of the PRLR promoter sequence that spans the STAT5 site and Sp1 and C/EBPβ sites are 5'GCATGCTGAAGAAAATCACTGTTTTGCC3’ (forward) and 5’ TGCACGAGGACATGAAGCTCCA 3’ (reverse).

### Statistical analysis

The significance of the differences among groups were determined by multiple Tukey's multiple-comparison test (one-way ANOVA) and significance of the differences between E_2_ treated versus control were determined by Student's *t*-test using the Prism software program (GraphPad Software, Inc, San Diego, California)
